# Atopic dermatitis and indoor use of energy sources in cooking and heating appliances

**DOI:** 10.1186/1471-2458-12-890

**Published:** 2012-10-22

**Authors:** Ana M Vicedo-Cabrera, Luís García-Marcos, Agustín Llopis-González, Ángel López-Silvarrey-Varela, Izaskun Miner-Canflanca, José Batlles-Garrido, Alfredo Blanco-Quiros, Rosa María Busquets-Monge, Carlos Díaz-Vazquez, Carlos González-Díaz, Antonio Martínez-Gimeno, Francisco Guillén-Grima, Alberto Arnedo-Pena, María Morales-Suárez-Varela

**Affiliations:** 1 Unit of Public Health, Hygiene and Environmental care, Department of Preventive Medicine, University of Valencia, Valencia, Spain; 2 Pulmology and Allergy Units, Virgen de la Arrixaca Children’s University Hospital, Murcia, Spain; 3 CIBER - Epidemiology and Public Health, Madrid, Spain; 4 Foundation María José Jove, A Coruña, Madrid, Spain; 5 Department of Paediatrics, Donostia Hospital, San Sebastián, Spain; 6 Department of Paediatrics, Torrecárdenas Hospital, Almería, Spain; 7 Department of Paediatrics, University of Valladolid, Valladolid, Spain; 8 Department of Paediatrics, del Mar Hospital, Barcelona, Spain; 9 Health Centre of Moreda, Health Service of Principado de Asturias, Madrid, Spain; 10 Department of Paediatrics, Basurto Hospital, Bilbao, Spain; 11 Department of Paediatric Pneumo-Allergy, 12 de Octubre Children’s Hospital, Madrid, Spain; 12 Section of Epidemiology, Centre of Public Health, Regional Ministry of Health, Castellón, Castellón, Spain; 13 Department of Health Sciences, Public University of Navarre, Navarre, Spain; 14 Centre for Public Health Research (CSISP), Valencia, Spain

**Keywords:** Atopic dermatitis, Electricity, Gas, Biomass, Indoor, Children

## Abstract

**Background:**

Atopic dermatitis (AD) prevalence has considerably increased worldwide in recent years. Studying indoor environments is particularly relevant, especially in industrialised countries where many people spend 80% of their time at home, particularly children. This study is aimed to identify the potential association between AD and the energy source (biomass, gas and electricity) used for cooking and domestic heating in a Spanish schoolchildren population.

**Methods:**

As part of the ISAAC (International Study of Asthma and Allergies in Childhood) phase III study, a cross-sectional population-based survey was conducted with 21,355 6-to-7-year-old children from 8 Spanish ISAAC centres. AD prevalence, environmental risk factors and the use of domestic heating/cooking devices were assessed using the validated ISAAC questionnaire. Crude and adjusted odds ratios (cOR, aOR) and 95% confidence intervals (CIs) were obtained. A logistic regression analysis was performed (Chi-square test, p-value < 0.05).

**Results:**

It was found that the use of biomass systems gave the highest cORs, but only electric cookers showed a significant cOR of 1.14 (95% CI: 1.01-1.27). When the geographical area and the mother’s educational level were included in the logistic model, the obtained aOR values differed moderately from the initial cORs. Electric heating was the only type which obtained a significant aOR (1.13; 95% CI: 1.00-1.27). Finally, the model with all selected confounding variables (sex, BMI, number of siblings, mother’s educational level, smoking habits of parents, truck traffic and geographical area), showed aOR values which were very similar to those obtained in the previous adjusted logistic analysis. None of the results was statistically significant, but the use of electric heating showed an aOR close to significance (1.14; 95% CI: 0.99-1.31).

**Conclusion:**

In our study population, no statistically significant associations were found between the type of indoor energy sources used and the presence of AD.

## Background

Atopic dermatitis (AD) is an inflammatory, chronically relapsing, pruritic skin disease causing loss of skin structure (lichenification). Its prevalence has considerably increased worldwide in recent years, mainly in children and young adults [1]. In fact, it is the most common inflammatory disease in these age groups, especially in industrialised countries where nowadays 25% of children have some allergic disorder. It is considered that this increase has occurred too quickly to be attributed to genetic factors in individuals alone or changes in diagnosis patterns of allergies. Thus, the emergence of new environmental risk factors and the loss of protective factors derived from a more traditional lifestyle are thought to play an essential role in the aetiology of atopic disorders [1-3].

Indoor environments are particularly relevant for the study of atopic diseases, especially in developed regions where many people spend most of their time at home [4]. Also, it should be noted that a huge number of well-known allergens coexist in this particular environment, such as molds, dust and air pollution. Regarding chemical elements, it is known that both combustion of heating/cooking systems and tobacco release toxic compounds to the air which considerably lower the quality of the indoor environment [5,6].

Pollutants are particularly harmful for children, especially in the postnatal period, as they are more sensitive to developing allergies. Their immune system is immature and it is more susceptible to be altered. Their skin is more fragile and permeable to external compounds and that makes them more vulnerable to infections and other disorders [2,4,7]. There is growing evidence that fuel combustion products act as adjuvants in the immune system and may lead to enhancement of allergic inflammation. It is shown that nitrogen dioxide (NO_2_) and particulate matter (PM_10_) bind to allergens in the air, increasing their allergenic power [6]. Also, diesel particulate matter and hydrocarbons triggers and increases the production of IgE [8]. Through these mechanisms, air pollutants may be an important contributor to the increasing prevalence and morbidity of allergic diseases.

More in detail, the exposure to atmospheric pollutants (in indoor and outdoor environment) has been extensively studied for its potential association with AD. NO_2_ has been reported to cause various effects in skin mucosa [9]. The presence of certain types of volatile organic compounds in indoor air was found to increase the presence of AD symptoms [10], and it was also shown that they can have an adverse effect on epidermal barrier function in humans [11]. However, the association between the use of domestic fuels and AD has not been widely studied to date; there remains lack of consensus. For instance, allergic sensitisation of the skin and allergic symptoms have been reported to appear when using gas and kerosene [6], yet other studies did not find this association [12]. Likewise, most of the evidences regarding this issue are based on studies performed in developing countries, where the poor quality of home devices constitutes a real risk for children’s respiratory health, especially the use of biomass fuels [13,14]. So, in some aspects, the conclusions drawn from these studies cannot be extrapolated to populations in a developed country where the use of more modern, efficient and insulated devices is highly spread [15].

So, the aim of this study was to explore the potential association between the presence of AD in 6- to 7-year-old children and energy sources used at home for heating/cooking in a developed country.

## Methods

### Study design, scope and study population

As part of Phase III of the ISAAC (International Study of Asthma and Allergies in Childhood) Project in Spain, a cross-sectional multicentre population-based study was conducted in 2002 and 2003 with 21,355 schoolchildren 6-7 years of age from 8 ISAAC Spanish centres (La Coruña, Asturias, Barcelona, Bilbao, Cartagena, Madrid, San Sebastián and Valencia).

Each ISAAC centre obtained authorisation from governing bodies and Parents Associations of each participant school after explaining both of them the aim of the study. Subsequently, parents were asked to sign a written consent and to fill in the Spanish version of the ISAAC phase III questionnaire. The Ethics Committee of 12 de Octubre Hospital (Madrid, Spain) approved the Spanish version of the ISAAC Phase III protocol and the operational plan.

### Questionnaire

A validated and standardised questionnaire [1], designed for ISAAC Phase III, was used to assess the prevalence of AD and exposure to environmental risk factors in the study population.

There is a consensus that a positive answer from parents to both of the following questions corresponds to the presence of AD symptoms [1,16,17]. 

“Has your child had an itchy rash that comes and goes in the last twelve months?”

“Has this itchy rash affected any of the following parts of the body: elbow creases, behind the knees, the front part of the ankles, under the buttocks, or around the neck, ears and eyes?”

The following data was collected from participants: sex, anthropometric characteristics (height and weight as reported by parents), and family environment (number of younger and older siblings, mother’s level of education, parents’ smoking habits, number smokers at home, and presence of pets (dogs or cats) in the last 12 months). BMI was calculated from the reported height and weight and children were classified according to the cut-off points reported by Cole et al [18]. Exposure to outdoor pollution was evaluated by asking about the intensity of the truck traffic on the street where children lived. Also, children were classified into three areas according to the geographical location of each ISAAC centre: Atlantic (La Coruña, Asturias, Bilbao, San Sebastián) which corresponds to the northern ISAAC centres, Continental (or central area) (Madrid), and Mediterranean or eastern centres (Cartagena, Valencia and Barcelona).

Information about the type of fuel was collected by asking which specific type of cooking/heating system was normally used at home. We distinguished between electric, gas and biomass systems for cooking purposes, and electric, gas/kerosene/paraffin and biomass heating. In Spain, biomass fuels are used in solid fuel stoves for cooking, and in charcoal burners and fireplaces for heating. So, the use of each type of energy sources for cooking purposes was evaluated according to positive or negative answer to the question: “In your house, what fuel is usually used for cooking?” Electricity (yes/no), gas (yes/no) and biomass (yes/no). In addition, a similar question was used for the evaluation of the type of heating system used at home: “In your house, what fuel is usually used for heating?” Electricity (yes/no), gas, kerosene, paraffin (yes/no) and biomass (yes/no). Also, new ones were created by combining these first six simple variables to estimate the joint contribution of both types of domestic systems (heating and cooking) depending on the fuel used. That is, we grouped those children who reported using or electric heating or electricity for cooking (‘Electric cooker or heating’ variable), those who used gas, kerosene or paraffin for heating or cooking purposes (‘Gas cooker or gas/kerosene/paraffin heating’ variable), and finally, we put together in a third group those who used biomass cooker or heating (‘Biomass cooker or heating’ variable).

In addition, we created a new variable called “Nature of energy source”, which had three categories: “clean”, those using electricity only; “dirty”, those with no electric systems, that is, a combined use of the other fuel types (gas/solid fuel and gas, kerosene, paraffin/wood, coal, diesel oil and other oil-based products); and “clean + dirty”, those that combine electricity with another or other type(s) of fuel.

### Statistical analysis

According to the previous criteria, prevalence of AD was calculated for all the study population and for each category of the descriptive analysis of the study population in terms of absolute and relative frequencies: by ISAAC centre and geographic area, general characteristics of the participants and the use of energy sources at home. Likewise, we performed an additional analysis in order to study the distribution of the use of each heating/cooking device according to the geographic area and the mother’s educational level, which are the two descriptive variables of the population that, *a priori*, would shown a strong linkage with the choice of the nature of domestic appliance.

For each variable, crude odds ratios (cORs) and 95% confidence intervals (CIs) were calculated. A logistic regression analysis was performed and the level of significance was determined by a Chi-square test (p-value < 0.05). Likewise, the association between the presence of AD and the domestic use of energy sources in cooking and heating appliances was evaluated using a multivariate logistic regression analysis. Firstly, a preliminary logistic model which included the geographical area and the mother’s level of education was used as adjusting variables. The rational of this approach was to sort out the influence of the conditional choice of a specific type of domestic device according to the background characteristics of the family. Then, a more complex model was designed by including variables representing the indoor and outdoor air quality (smoking habits of parents and truck traffic), individual risk factors (sex, BMI, number of siblings, mother’s level of education) and characteristics of the area of residence (geographical area). These variables were selected according to the previous knowledge about their possible role as confounding variables, and in the light of the results of preliminary descriptive analysis. We assessed the association in terms of adjusted odds ratios (aORs) and 95% confidence intervals. The level of significance was determined by Chi-square test (p-value < 0.05). All variables were treated as categorical, except for BMI, which was treated as continuous. The statistical analysis was performed using the Stata v11 statistical software (Stata Corp., College Station, TX).

## Results

### Study participation and AD distribution

The study sample included a total of 1,276 cases of AD, with an overall AD prevalence of 6.0% in the whole study population. A p-value < 0.01 was obtained when comparing prevalence across the different ISAAC centres: San Sebastian showed the highest AD prevalence (8.2%), while Barcelona had the lowest (3.9%) (Table 1). There were also significant differences between the geographic areas, where the Atlantic obtained the highest percentage (7%), followed by the Continental (6.0%) and finally the Mediterranean area with 4.9% (p-value < 0.001).

**Table 1:** Study population and atopic dermatitis prevalence for each ISAAC area/centre

**AREA/CENTRE**	**STUDY POPULATION N**	**ATOPIC DERMATITIS**	
		**N**	**%**
Atlantic Area	9 990	694	7.0
A Coruña	3 017	220	7.3
Asturias	3 027	198	6.5
Bilbao	3 046	202	6.6
San Sebastián	900	74	8.2
Continental Area - Madrid	2 344	141	6.0
Mediterranean Area	9 021	441	4.9
Barcelona	2 902	113	3.9
Cartagena	2 726	129	4.7
Valencia	3 393	199	5.9
Total	21 355	1 276	6.0

### General characteristics of individuals with AD

As it is shown in Table 2, the prevalence of AD was slightly higher in females than in males, with a cOR value over 1 but not stastically significant. The highest prevalence of AD was found in the obese children, with a significant cOR value of 1.34 (95% CI: 1.14-1.58). Furthermore, the highest prevalence of AD was found among children whose mothers had professional degrees and college education, and among those children who had younger siblings at home, but with cOR values near significance.

**Table 2:** Characteristics of the study population as a function of the presence of atopic dermatitis (AD)

	**AD**		**cOR**	**95% CI**
	**N**	**%**		
Sex				
Male	608	5.7	1.00 (ref)	
Female	667	6.2	1.10	0.98-1.23
BMI categories				
Underweight	90	5.2	0.91	0.72-1.15
Normal weight	505	5.7	1.00 (ref)	
Overweight	242	5.8	1.02	0.87-1.19
Obese	226	7.6	1.34	1.14-1.58*
Mother’s level of education				
No education	50	5.5	1.00 (ref)	
Primary education	307	5.1	0.92	0.68-1.26
Secondary education	462	5.9	1.06	0.79-1.44
Tertiary education	457	6.9	1.26	0.94-1.71
Older siblings				
No	645	6.0	1.00 (ref)	
Yes	593	5.9	1.00	0.89-1.12
Younger siblings				
No	745	5.8	1.00 (ref)	
Yes	480	6.3	1.10	0.98-1.24
No. of cigarettes/day -mother				
No smoker	735	5.8	1.00 (ref)	
1-5	86	5.2	0.89	0.71-1.12
6-10	161	6.3	1.10	0.91-1.30
11-20	227	6.7	1.18	1.01-1.37*
>20	38	7.8	1.37	0.98-1.93
No. of cigarettes/day -father				
No smoker	703	6.0	1.00 (ref)	
1-5	68	5.9	0.98	0.76-1.26
6-10	76	4.1	0.68	0.53-0.86
11-20	240	6.3	1.05	0.90-1.22
>20	93	7.4	1.26	1.00-1.57*
No. of smokers at home				
0	559	5.8	1.00 (ref)	
1-2	655	6.3	1.09	0.97-1.23
>2	62	5.1	0.87	0.66-1.14
Cat or dog ever				
No	1 056	6.0	1.00 (ref)	
Yes	209	6.1	1.04	0.89-1.21
Truck frequency				
Never	189	5.7	1.00 (ref)	
Seldom	560	5.6	0.98	0.83-1.17
Frequently through the day	431	6.4	1.13	0.95-1.34
Almost the whole day	84	8.4	1.53	1.17-2.00*
Area				
Mediterranean	441	4.9	1.00 (ref)	
Continental	141	6.0	1.25	1.02-1.51*
Atlantic	694	7.0	1.45	1.28-1.64*

*cOR*: crude odds ratio. *BMI*: body mass index.

*Chi-squared test. p ≤ 0.05.

Regarding the smoking habit in the family environment, it was found that the prevalence of AD increases with the number of cigarettes/day among those whose mother smoked. However, this pattern was not clear for children whose father smoked. It should be noted that the prevalences of AD in children with non-smoker parents were not the lowest ones compared to the percentages obtained in each cigarette consumption category. However, statistically significant cOR were found in the ‘11-20’ category for mothers (1.18 (95% CI: 1.01-1.37) and in ‘>20’ for fathers (1.26 (95% CI: 1.01-1.37)).

Number of smokers at home and the presence of pets in the last 12 months did not show statistically significant results. However, a highly significant cOR of 1.53 (95% CI: 1.17-2.00) was obtained for those children whose parents described the intensity of truck traffic as ‘almost the whole day’, with an AD prevalence of 8.4%.

### Use of the different types of cooking and heating appliances

In Table 3, it is shown the percentage of reported use of each domestic appliance according to the two characteristics of the population that would influence its choice: firstly, we distinguished between the different geographic areas in order to evaluate in a general way the climate and the habits of the population in each area. In this case, we obtained great differences between the percentages of use of each domestic appliance in the three areas except for biomass cooker. Gas cooker (79.7%), and electric heater (55.2%) were highly used in the Mediterranean area, gas heater (64.9%) in the Continental one, and finally, electric cooker (62.0%) and biomass heater (18.0%) in the Atlantic area. Likewise, we considered mother’s educational level as a proxy of the familiar social status and, also, this variable would show the degree of awareness about the risk that certain fuels represent for health. Again, statistically significant differences were obtained, except for the use of biomass cooking devices. Gas cooker (67.9%) and electric heater (48.6%) were mostly used by families whose mother had a primary education, whereas the percentage of use of electric cooker (52.9%), gas (53.4%) and biomass (13.6%) heating appliances was higher in the category of mothers with the highest educational level. In both cases, it should be noted that each group of percentages (for cooking and heating devices) cannot sum 100% in each category because the use of one kind of device for cooking or heating is not exclusive. That is, for example, a family can use an electric heater but also a biomass stove.

**Table 3:** Distribution of the use of each heating/cooking device according to the geographic area and the mother’s educational level

	**Cooking**			**Heating**		
	**Electric**	**Gas**	**Biomass**	**Electric**	**Gas/kerosene/paraffin**	**Biomass**
Area						
Mediterranean	1 929 (21.4)	7 190 (79.7)	27 (0.3)	4 975 (55.2)	3 549 (39.3)	257 (2.9)
Continental	852 (36.4)	1 481 (63.2)	7 (0.3)	504 (21.5)	1 522 (64.9)	198 (4.5)
Atlantic	6 196 (62.0)	3 920 (31.1)	50 (0.5)	3 087 (30.9)	4 786 (47.9)	1 792 (18.0)
Mother’s level of education						
No education	222 (24.6)	555 (61.5)	3 (0.3)	335 (37.1)	306 (33.9)	49 (5.4)
Primary education	1 988 (33.3)	4 052 (67.9)	34 (0.6)	2 897 (48.6)	2 312 (38.8)	539 (9.0)
Secondary education	3 269 (41.6)	4 722 (60.0)	28 (0.4)	3 221 (40.9)	3 707 (47.1)	761 (9.7)
Tertiary education	3 498 (52.9)	3 262 (49.3)	19 (0.3)	2 113 (31.9)	3 532 (53.4)	898 (13.6)

N (%): absolute and relative frequency of use of each type of cooking/heating appliance in each category of area and mother’s level of education.

The distribution of AD prevalence between the different variables representing the energy sources regularly used at home for cooking and heating are shown in Table 4. Regarding the six original variables, it was found that the use of biomass systems gave the highest cORs, especially in the case of biomass cookers. The cOR was 1.17 (95% CI: 0.98-1.39) for the use of biomass heating was close to significance, but only the use of electric cooker showed a significant cOR of 1.14 (95% CI: 1.01-1.27) for AD development.

**Table 4:** Estimation of the crude and adjusted odds ratios (cORs and aORs) for the types of energy source used at home as a function of the presence of atopic dermatitis (AD)

	**AD**		**cOR**	**aOR 1**	**aOR 2**
	**N**	**%**			
Electric cooker					
No	702	5.7	1.00 (Ref)	1.00 (Ref)	1.00 (Ref)
Yes	574	6.4	1.14 (1.01-1.27)*	0.96 (0.85 - 1.08)	0.94 (0.81-1.09)
Gas cooker					
No	547	6.2	1.00 (Ref)	1.00 (Ref)	1.00 (Ref)
Yes	729	5.8	0.92 (0.82-1.04)	1.10 (0.97-1.25)	1.10 (0.95-1.28)
Biomass cooker					
No	1 268	6	1.00 (Ref)	1.00 (Ref)	1.00 (Ref)
Yes	8	9.5	1.66 (0.80-3.45)	1.66 (0.80-3.44)	1.51 (0.60-3.81)
Electric heating					
No	763	6	1.00 (Ref)	1.00 (Ref)	1.00 (Ref)
Yes	513	6	1.00 (0.89-1.13)	1.13 (1.00-1.27)*	1.14 (0.99-1.31)
Gas/kerosene/paraffin heating					
No	695	6.1	1.00 (Ref)	1.00 (Ref)	1.00 (Ref)
Yes	581	5.9	0.97 (0.87-1.09)	0.92 (0.82-1.04)	0.91 (0.80-1.04)
Biomass heating					
No	1 123	5.9	1.00 (Ref)	1.00 (Ref)	1.00 (Ref)
Yes	153	6.8	1.17 (0.98-1.39)	1.02 (0.82-1.22)	1.02 (0.82-1.25)
Electric cooker or heating					
No	405	5.6	1.00 (Ref)	1.00 (Ref)	1.00 (Ref)
Yes	871	6.2	1.10 (0.97-1.24)	1.05 (0.93-1.19)	1.27 (0.94-1.72)
Gas cooker or gas/kerosene/paraffin heating					
No	311	6.2	1.00 (Ref)	1.00 (Ref)	1.00 (Ref)
Yes	965	5.9	0.95 (0.83-1.08)	1.04 (0.91-1.19)	1.01 (0.86-1.19)
Biomass cooker or heating					
No	1 121	5.9	1.00 (Ref)	1.00 (Ref)	1.00 (Ref)
Yes	155	6.8	1.16 (0.98-1.38)	1.02 (0.85-1.22)	1.00 (0.82-1.24)
Nature of energy source					
Clean	186	6.4	1.00 (Ref)	1.00 (Ref)	1.00 (Ref)
Clean + dirty	799	6.2	0.96 (0.82-1.14)	0.99 (0.84-1.17)	1.18 (0.80-1.76)
Dirty	291	5.4	0.83 (0.69-1.00)	0.88 (0.73-1.07)	0.89 (0.56-1.42)

*cOR*: crude odds ratio. *aOR*: adjusted odds ratio.

*Chi-squared test. p ≤ 0.05.

1) Adjusted by: mother’s educational level, geographic area.

2) Adjusted by: gender, BMI, siblings, smoking habit (mother and father), mother’s educational level, geographic area, truck traffic.

Again, among the combined variables, the use of a biomass cooker or biomass heating or both (‘Biomass cooker/heating’) showed the highest cOR value (1.16; 95% CI: 0.98-1.38), followed by ‘electric cooker/heating’ variable (1.10 (95% CI: 0.97-1.24) which were close to significance. Regarding the variable ‘Nature of energy source’, all cOR values were smaller than one, but non-significant. It should be noted that nobody in the study population used biomass as unique source for heating and cooking purposes That means the category of ‘dirty’ only includes children whose houses only had gas/kerosene/paraffin heating and gas cooker.

When the geographical area and the mother’s educational level were included in the logistic model, the obtained aOR values differed moderately from the initial cORs, with a non-clear pattern, except for the ‘biomass cooker’ and the ‘Nature of energy source’ variable. However, only the electric heating obtained a significant aOR (1.13; 95% CI: 1.00-1.27).

Finally, the more complex logistic model, which included all selected confounding variables, showed aOR which were very similar to the values obtained in the previous analysis. None of the aOR values was statistically significant, but it should be noted that again the use of electric heating showed an aOR close to significance (1.14; 95% CI: 0.99-1.31).

## Discussion

The quality of household environment is rapidly gaining importance as a public health issue worldwide, especially regarding respiratory diseases and allergic disorders. In fact, the specific climatic characteristics, the reduced ventilation, the presence of identified indoor allergens such as mold, dust mites, animal dander and cockroaches, along with the ambient concentrations of pollutants from the combustion of domestic devices and tobacco, make indoor environment a mixture of risk factors for the development of allergies [19]. A large number of studies have been performed in the last decades dealing with the indoor air pollution, and most of them aimed to identify its possible association with health outcomes in children. In the case of the present study, we aimed to identify the potential association between the presence of AD in 6-to-7-years old population and the use of electric, gas and biomass heating and/or cooker at home. Unfortunately, our results failed to show a clear relationship between them, which will be discussed in next lines.

Although indoor air pollution is a huge public health concern, little research has been conducted on the relationship between domestic fuels and AD [6,12]. Respiratory disorders, asthma and rhinitis are the main studied outcomes because the inhalation of pollutants is considered to be the main source of exposure. However, it should be taken into account that the skin is the first human body’s main line of defence against external factors, and if altered, its functional barrier would be deteriorated facilitating the entrance of chemical and biological elements into the internal compartments [1]. Besides that, most of the evidences are based on studies that have been carried out in developing countries where living conditions differ from those in Spain [20,21]. As it is said above, our study population belongs to a developed country where the technology advances have extensively released new gas modern heating appliances. These are provided with closed conductions for residual gases of the combustion process, more efficient and insulated devices and cleaner installations [15]. So, they are expected to be safer in terms of toxicological risk factors comparing to this kind of heaters used in developing countries.

On the other hand, the strongest evidences are those that link the use of biomass domestic devices and the health outcomes in childhood [4,20,22]. The World Health Organization stated that in 2002, Sub-Saharan Africa and South-East Asia led with 396 000 and 483 000 deaths due to indoor smoke, respectively [23,24]. It is established that the combustion of biomass fuels releases more polluting substances to indoor environments than gas burners, so its potential toxicity is higher too [20,24]. Fortunately, in Spain, biomass is much less frequently used than either gas or electricity as a domestic fuel. In fact, the use of biomass fuels is associated with more rural and poorer areas; specifically such fuels are burned in fireplaces, firewood stoves and cookers, charcoal burners and other simple heating devices, and these are no longer widely used in our country. Actually, in our study population, the number of biomass users was very limited and nobody used biomass as unique energy source at home.

Although the present study did not obtain sufficient evidences to conclude that the use of combustion fuels at home could be associated with the development of AD, the role of electricity as a ‘clean’ domestic energy source is not clear. An almost statistically significant association was found for the use of electric heater despite being considered a clean source of energy in terms of releasing polluting substances into the air [15]. As it is said above, the toxicological hypothesis should be put aside in the present study due to the developed characteristics of the studied population. Otherwise other possible reasons should be considered: firstly, it would be necessary to assess the potential effect of electromagnetic fields generated by electrical appliances on children′s immune systems and their effect on AD development in particular. In fact, the presence of electromagnetic fields has been shown to affect the immune system [25] and may, therefore, be involved in some way in the aetiology of AD [26]. Secondly, these results might be in line with the “hygiene theory”; it hypothesises that the non-exposure to risk factors during childhood and development of infections results in a lack of exposure to the necessary stimuli to help the immune system mature with the following inception of allergic disorders [2,27]. And finally, it should be noted that the nature and efficiency of heating systems may influence the development of allergic disorders by modifying the climate indoors [28]. For example, it has been shown that the environmental humidity can alter the mast cells increasing the histamine content in the dermis, which results in the modification of the allergic process [29]. In this case, it is known that the use of electric heaters, including air conditioning systems, which also act as emitters of hot air, may contribute to dry the indoor environment. By the same token, changing indoor climate due to the use of heaters at home could modify the effect of other allergens [30,31].

As expected, the present work showed that the mother’s level of education and the geographic area influence the distribution of the percentages of use of each domestic device. Likewise, it seems that the choice of a specific fuel did not follow the same pattern for cookers and heating appliances. For instance, it is shown that users of electric cooker did not use the same energy source for heating purposes, as it could be observed with the low number of subjects included in the ‘nature of energy source’ variable. This could be a reason why it was difficult to obtain the ‘pure’ contribution of each fuel to the development of AD.

### Study limitations

Although the ISAAC questionnaire has been validated and it has been widely used in epidemiological studies involving allergy in children, the presence of AD or not in individuals is based on parents’ self-reports through two questions. This evidently shows the typical limitations in verifying the diagnoses of AD [1]. Also, the ISAAC phase III in Spain did not collect information about parental history of allergy. Likewise, it should be taken into account that the results regarding the environmental risk factors could be influenced by the presence of reporting bias, as all self-reported exposure questionnaires show.

In order to assess the real role of domestic fuels used at home in AD development, other variables should be considered: whether there are fume extraction systems and, if so, their characteristics, the home size, each cooking/heating system type, fuel quality, etc. The amount of time a child spends at home, especially near combustion systems, should also be assessed. It is also important to remember that despite the general belief that the more ventilation, the lower exposure to indoor pollution, it also implies more exposure to outdoor air; that is, external environmental pollutants may also enter homes. This means that, in practice, it is very difficult to determine children’s exact level of exposure to the various fuels or residues released at home by indoor systems. On the other hand, due to lacking data, this study has not attempted to assess the degree of exposure to fuels in closed places other than their home where children spend much of their time (i.e., schools), the presence of other allergens in domestic environments (i.e., mould, dust, mites, etc.) [32,33], dietary habits [34] or alternative variables which represent in a more accurate way the social status of the family. Accordingly, it should be taken into account the possible presence of residual confounding in the present analysis.

## Conclusions

The present study did not show evidences of a clear association between the use of any particular domestic device for heating or cooking purposes and the presence of AD in this particular children’s population. The risk assessment of indoor environment constitutes a highly complex issue, and its conclusions should be carefully regarded, taking into account specific characteristics of the targeted population.

## Abbreviations

AD: Atopic dermatitis; NO_2_: Nitrogen dioxide; PM_10_: Particulate matter; ISAAC: International Study of Asthma and Allergies in Childhood; BMI: Body mass index; cORs: Crude odds ratios; CIs: Confidence intervals; aORs: Adjusted odds ratios.

## Competing interests

The authors declare that they have no competing interests.

## Authors’ contributions

LGM, ALG, ALSV, IMC, JBG, ABQ, RMBM, CDV, CGD, AMG and MMSV have made substantial contributions to conception and design and acquisition of data. AVC, LGM and MMSV have participated in the analysis and interpretation of data. FGG and AAP contribute with the data collection and evaluation of the manuscript. AVC, LGM and MMSV have been involved in drafting the manuscript or revising it critically for important intellectual content; and AVC, LGM, ALG, ALSV, IMC, JBG, ABQ, RMBM, CDV, CGD, AMG and MMSV have given final approval of the version to be published. All authors read and approve the final manuscript.

## Pre-publication history

The pre-publication history for this paper can be accessed here:

http://www.biomedcentral.com/1471-2458/12/890/prepub
